# Evaluation of the concentration of the coat of dogs aeroallergens (Canis lupus familiaris) and the dust from families of children with asthma and or allergic rhinitis

**DOI:** 10.1186/1939-4551-8-S1-A171

**Published:** 2015-04-08

**Authors:** Marconi Rodrigues De Farias, Michelle Barbosa, L Karla Arruda, Nelson Rosario Filho

**Affiliations:** 1 School of Agricultural Sciences and Veterinary Medicine, Pontifícia Universidade Católica Do Paraná, Brazil; 2 School of Medicine, University of São Paulo, Ribeirão Preto, Brazil; 3 Federal University of Parana, Brazil

## Background

Allergens from house dust mites are perennial and also have enzymatic nature. They are commonly found in bedding, mattresses, pillows, bedroom floor and living room. In addition, they are often associated with sensitization and intensification in the symptoms of allergic rhinitis and asthma for susceptible individuals. The aim of this study was to evaluate the concentrations of Der p 1, Der f 1, Blo t 5, Can f 1 and Fel d 1 in the coat of dogs and spread throughout the environment to check if dogs can serve as a reservoir of allergens for the space as well as being able to trigger allergic reactions in their owners and other individuals.

## Methods

For this end, it was selected houses of 53 children with symptoms of allergic rhinitis or asthma, where 32 lived with dogs in their homes (group 1) and 21 do not live with dogs (group 2). Samples of household dust and fur of dogs were collected to evaluate the levels of allergens by ELISA specific allergen method. All the data were statistically analyzed, considering the minimum significance level of 5%.

## Results

In relation to the dog’s coat, the average concentrations for Der p 1 (0.4 µg.g^-1^), Der f 1 (0.3 µg.g^-1^) and Blo t 5 (0.3 µg.g^-1^) were lower than those for animal allergens, Can f 1 (3.3 µg.g^-1^) (p <0.001) and Fel d 1 (1.3 µg.g^-1^). In the environment, the most common allergen Der p 1 was found at a concentration of 118.1 µg.g^-1^ in bedding (p <0.001), 19.0 µg.g^-1^ in mattress and 1.1 µg.g^-1^ on the ground. Animal allergens were found in equal proportion on environments with and without dogs (p> 0.05), the concentration of Can f 1 and Fel d 1 was higher in environments with dogs (p <0.001).

## Conclusions

It is correct to assure that the coat of dogs can carry and spread to the environment mainly animal allergens and also can carry mite allergens for about 1/3 of the time, but in concentrations with no sensitized, which do not contribute significantly to their environmental existence.

